# 5-hydroxymethylcytosines from circulating cell-free DNA as noninvasive prognostic markers for gastric cancer

**DOI:** 10.1371/journal.pone.0335654

**Published:** 2025-11-20

**Authors:** Yingli Fu, Donghui Cao, Yanhua Wu, Zhifang Jia, Yangyu Zhang, Chenhao Fu, Xueyuan Cao, Jing Jiang

**Affiliations:** 1 Division of Clinical Epidemiology, The First Hospital of Jilin University, Changchun, Jilin Province, China; 2 Department of Epidemiology and Biostatistics, School of Public Health, Jilin University, Changchun, Jilin Province, China; 3 Department of Gastric and Colorectal Surgery, General Surgery Center, The First Hospital of Jilin University, Changchun, Jilin Province, China; University of Florida College of Public Health & Health Professions, UNITED STATES OF AMERICA

## Abstract

**Background:**

Prognostic assessment plays a crucial role in guiding clinical management and treatment decisions for gastric cancer patients. The enrichment characteristics of 5-hydroxymethylcytosine (5hmC) in circulating cell-free DNA (cfDNA) has emerged as potential prognostic epigenetic markers.

**Methods:**

Using 5hmC-Seal combined with next-generation sequencing (NGS), we profiled the genome-wide distribution of 5hmC in plasma cfDNA samples from 51 gastric cancer patients. Prognostic biomarkers were selected via random survival forest and Cox proportion hazards models, and a prognostic model was subsequently constructed.

**Results:**

Seven prognostic biomarker genes were identified, and the 7-gene prognostic model demonstrated a concordance index (C-index) of 0.892 (95% CI = 0.786–0.998). Patients in the high risk group had a significantly worse overall survival (OS) than those in low-risk group (log-rank P = 0.00012). When the cfDNA 5hmC risk-score was integrated with the traditional clinical characteristics, the C-index increased from 0.819 (95% CI = 0.727–0.911) to 0.904 (95% CI = 0.853–0.955). Multivariate analysis adjusted for age, TNM stage, and chemotherapy confirmed that a high risk-score of cfDNA 5hmC model was an independent predictor of poor OS (hazard ratio [HR]=27.47, 95% CI = 3.28–230.25).

**Conclusion:**

cfDNA 5hmC serves as an effective prognostic biomarker with high predictive value for the long-term survival in postoperative gastric cancer patients.

## 1. Introduction

Gastric cancer (GC) is the fifth most diagnosed malignancy worldwide, with nearly 1 million estimated new cases annually [1]. Despite advances in surgical techniques and chemoimmunotherapy, the global 5-year survival rate was remains only 20–40% [2]. Prognostic assessment plays a crucial role in guiding clinical management and therapeutic strategies for GC patients. Circulating cell-free DNA (cfDNA), a kind of non-invasive diagnostic and prognostic biomarker, has emerged as a powerful tool to overcome tumor heterogeneity. Its utility in cancer detection and prognosis has been demonstrated through the quantification of tumor-derived single-nucleotide variants (SNVs), copy number alterations (CNAs), and epigenetic aberrations [3–6].

Stable and homogeneous DNA epigenetic alterations are recognized as promising targets for biomarker development. 5-Hydroxymethylcytosine (5hmC), an intermediate product of DNA demethylation, is generated through the oxidation of 5-methylcytosine (5mC) by ten-eleven translocation (TET) enzymes [7]. This epigenetic modification is predominantly enriched in functionally active genomic regions, including enhancers, promoters, and gene bodies, where its levels exhibit a positive correlation with transcriptional activity [8]. Emerging evidence indicates that dynamic 5hmC alterations are associated with cancer initiation, progression, metastasis, and prognosis across multiple malignancies, including gastric cancer [9–13]. Notably, 5hmC profiling has recently gained attention as a novel prognostic epigenetic marker in oncology research.

To detect 5hmC enrichment in cfDNA, the 5hmC-Seal method coupled with next-generation sequencing (NGS) has been widely utilized in cancer research. This approach enables sensitive detection of trace 5hmC modifications and has demonstrated potential as both a diagnostic and prognostic marker across multiple cancer types [14–16]. In our previous study, we reported a gastric cancer diagnostic biomarker model based on the cfDNA 5hmC signatures of 50 gastric cancer patients and 50 matched non-cancer controls, and the biomarker model showed great diagnostic value [17]. Since biomarkers can not only signal disease occurrence but are also used to predict disease prognosis, this study conducted regular follow-up on these 50 gastric cancer patients plus one additional gastric cancer patient who was excluded from the diagnostic analysis due to incomplete pairing (totaling 51 gastric cancer patients). Based on the cfDNA 5hmC sequencing data, clinical characteristics, and follow-up data of these 51 gastric cancer patients, this study screened potential prognostic biomarkers for gastric cancer to construct a prognosis predictive model. Furthermore, it analyzed whether incorporating this predictive factor enhances the accuracy of prognostic predictions when combined with typical clinical characteristics.

## 2. Materials and methods

### 2.1 Patients and Follow-up

A total of 51 patients with histologically diagnosed GC) who underwent radical gastrectomy at the Department of Gastric and Colorectal Surgery in the First Hospital of Jilin University (Changchun, China) from 01/01/2018 to 01/12/2021 were recruited in this cohort study.These patients were derived from a gastric cancer patient cohort established since 01/01/2018. From 01/06/2023 to 30/04/2024, we retrospectively reviewed this patient cohort database to obtain their general demographic, clinicopathological characteristics, and follow-up data.

Patients included were first-time diagnosed with GC and received tumor resection; the diagnosis was confirmed by histopathological examination of tumor tissue, and all subjects had peripheral blood collected without any radiotherapy, chemotherapy, or immunotherapy before any tumor resection treatment. All patients were accounted for at the first follow-up. The exclusion criteria were as follows: ① patients with any other tumors, ② patients with distant metastasis. Clinical, pathological, and treatment data of GC patients were obtained from medical records.

Follow-up was implemented at 3 months, 6 months, 12 months and annually afterwards until death or the end of the follow-up. Information on general status and postoperative chemotherapy were collected during each follow-up. If the patients had died, the date of death and potential cause were recorded. The duration from the date of surgery to the date of death or the last successful interview date was defined as the survival time. If the patient was lost to follow-up, survival time was defined as the duration from the date of surgery to the date of the last successful interview. All patients were followed up for a minimum of 30 months.

This study was reviewed and approved by the Ethics Committee at The First Hospital of Jilin University (NO. 19K042-001). Written informed consent was obtained from each participant, and biospecimens were collected as approved by the Institutional Review Boards (IRBs) responsible at the First Hospital of Jilin University.

### 2.2 Sample preparation and 5hmC-Seal Sequencing

Approximately 10 ml peripheral blood samples from each participant were collected in PAXgene Blood ccfDNA Tubes according to the manufacturer’s protocol (Qiagen, Hilden, Germany). Plasma was separated from the whole blood samples by centrifuging twice. cfDNA was isolated from the clean plasma using the QIAamp Circulating Nucleic Acid Kit (Qiagen, Germantown, MD, USA) following the manufacturer’s protocol.

The cfDNA was prepared and ligated with Illumina compatible adaptors and purified on a Micro Bio-Spin 30 Column (Bio-Rad, Hercules, CA, USA). The cfDNA concentration of each library was measured with a Qubit fluorometer (Life Technologies, Carlsbad, CA, USA), and sequencing was performed on the Nova6000 platform. Samples that passed the quality test were transferred to the following sequencing steps. The raw 5hmC-Seal data were trimmed using Trimmomatic software (version 0.36) and checked for quality by Bioanalyzer dsDNA. A high-sensitivity assay (Agilent Technologies, Santa Clara, CA), followed by alignment to the human genome reference (hg19), was conducted by Bowtie2 (version 2.2.6). DeepTools (version 3.5.1) was used to conduct a series of processing steps on the sequencing result files and calculate the profiling. More details about the cfDNA quality test, 5hmC-Seal library preparation and sequencing and the data processing pipelines were described in previous publications [17]. The 5hmC-seal count data were normalized by correcting for sequencing depth and library size using DESeq2 (version 1.12.3).

The 5hmC read count per million mapped reads of the gene body (from TSS to TTS) was calculated as the 5hmC level of each gene. 5hmC modification of the gene body region was used as marker region of each gene supported by previous published results [17–19]. The online NIH/DAVID tool (https://david.ncifcrf.gov/tools) and Wei Sheng Xin (https://www.bioinformatics.com.cn/) were used to analyze the functional enrichment in Gene Ontology (GO) and Kyoto Encyclopedia of Genes and Genomes (KEGG) databases. The P values of the enriched terms were corrected by the Holm Bonferroni method.

### 2.3 Statistics methods

Categorical variables are presented as n (%). Mean±standard deviation (SD) presented for normally distributed continuous variables, while median [interquartile range (IQR)] was given to those with non-normally distributed continuous variables.

Univariate Cox regression analyses were performed to select prognostic associated marker genes one by one with a P-value <0.05. RSF model was built by ensemble binary trees grown on bootstrapped samples to select the most important variables that are associated with time to death by R randomSurvivalForest package. Bootstrapping and random node splitting were used to grow an ensemble of binary trees to form the RSF model. We used 1000 trees to construct our models with the square root of the number of predictors sampled at each split time. When constructing a bootstrap sample in the ensemble, certain samples are left out. The average of the out-of-bag (OOB) performance measures can then be used to evaluate the predictive performance of the entire ensemble. Approximately 37% of the dataset, classified as OOB instances, were excluded from each tree’s training set and used for validation purposes.

VIMP was obtained by measuring the decrease in prediction accuracy using out-of-bag data which were not used for building trees each time. At each iteration, the variable with least importance score was removed and a model was rebuilt using the remaining variables, and the prediction error rate of the model was recorded. The process was repeated until all variables were removed. Then the variables in smallest prediction error model was selected as potential markers.

To evaluate how each of the individual analytes selected by RSF was linked to time to death, we used stepwise Cox proportional hazards regression model to construct the predictive model with selected variables at final by R survival package. To quantify the risk-scores for the final prognostic model, we employed the following formula according to the “risk” type of Coxph function of R survival package.:β1 × gene1 + β2 × gene2 + … + βp×genep, where βp is the coefficient for the kth marker gene from the final multivariable Cox proportional hazards model, and genep is the normalized 5hmC level of the kth marker gene. Seven genes were analyzed using a multivariate Cox proportional hazards model. Kaplan-Meier plot with log-rank test was performed to examine the OS difference across different risk-score group.

Model discrimination was measured by the Harrell’s C-index and tAUC, which corresponds to the proportion of random pairs of cases where one patient is alive and one dead at a specified time point where the model has correctly ordered their probability of survival having weighted for censoring [20], with R pec package. Model calibration was measured by the calibration plot using R rms package. Complex model including risk-score of cfDNA 5hmC markers and clinical characteristics was further constructed to evaluate the incremental value of cfDNA 5hmC markers. The incremental value of cfDNA 5hmC risk-score was assessed using both category-free net reclassification improvement (NRI) and integrated discrimination improvement (IDI) by R survival package. All statistical analyses were performed with R version 3.5.2 (http://www.r-project.org/). This study complies with the Transparent Reporting of a multivariable prediction model for Individual Prognosis Or Diagnosis (TRIPOD) guideline statement [21].

## 3. Results

### 3.1 Patient characteristics

This study enrolled 51 GC patients who underwent curative tumor resection and completed initial postoperative follow-up (Table 1).

**Table 1:** Demographics and clinical characteristics of the study population.

Characteristics	Group	n(%)
Sex	Male	35(68.6)
	Female	16(31.4)
Depth of invasion	T1	9(17.6)
	T2	6(11.8)
	T3	21(41.2)
	T4	15(29.4)
Lymph metastasis	N0	17(33.3)
	N1-N3	34(66.7)
TNM stage	Stage I	11(21.6)
	Stage II	15(29.4)
	Stage III	25(49.0)
Tumor size	<0.5 cm	34(66.7)
	≥0.5 cm	17(33.3)
Vascular invasion	Positive	36(70.6)
	Negative	15(29.4)
Neural invasion	Positive	27(52.9)
	Negative	24(47.1)
Lauren classification	Intestinal	18(35.3)
	Diffuse	12(23.5)
	Mixd	21(41.2)
Chemotherapy	Yes	23(45.1)
	No	28(54.9)

The cohort had a median age of 60.3 ± 7.3 years (range: 41–77 years), and 68.6% (n = 35) participants were male. Pathological assessment revealed vascular invasion in 70.6% of cases and neural invasion in 52.9%. Adjuvant chemotherapy was administered to 45.1% of patients. According to the American Joint Committee on Cancer/ Union for International Cancer Control (AJCC/UICC) 8th Edition Gastric Cancer Staging System, 21.6% (n = 11) of patients were classified as TNM stage I. During the follow-up period (ended on April 30, 2024), 15 patients (29.4%) died.

### 3.2 Identification and functional annotation prognosis-associated genes

**Fig 1:**
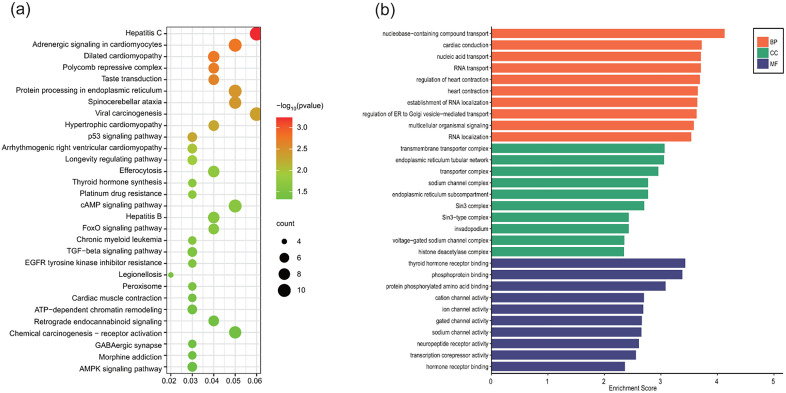
Functional enrichment analysis of GC patient prognosis associated genes (a. Top 30 enriched KEGG pathways. **b.** Top 10 enriched GO terms of each function module. BP, biological process, CC, cellular component, MF, molecular function).

We mapped 5hmC-modified gene bodies to the adult gastric genome (which was defined in ENCODE GENCODE, http://genome.ucsc. edu/ENCODE/) and identified 19,100 annotated genes. Univariate Cox regression analysis revealed 445 candidate genes significantly associated with overall survival (P < 0.05). To investigate the biological relevance of these prognosis-linked hydroxymethylation changes, we performed functional enrichment analysis using Kyoto Encyclopedia of Genes and Genomes (KEGG) and Gene Ontology (GO) databases. The results of the KEGG analysis showed that these genes were mainly enriched in tumor-related protein processing in the endoplasmic reticulum, the cMAP signaling pathway, ATP-dependent chromatin remodeling, and the p53 signaling pathway (Fig 1a). GO analysis highlighted molecular functions and cellular components associated with: nucleic acid transport, transmembrane transporter complex, and phosphoprotein binding functions (Fig 1b). More details of the enriched terms are listed in Supplemental table 1 (KEGG) and Supplemental table 2 (GO).

### 3.3 Prognostic marker genes selection and model construction

**Fig 2:**
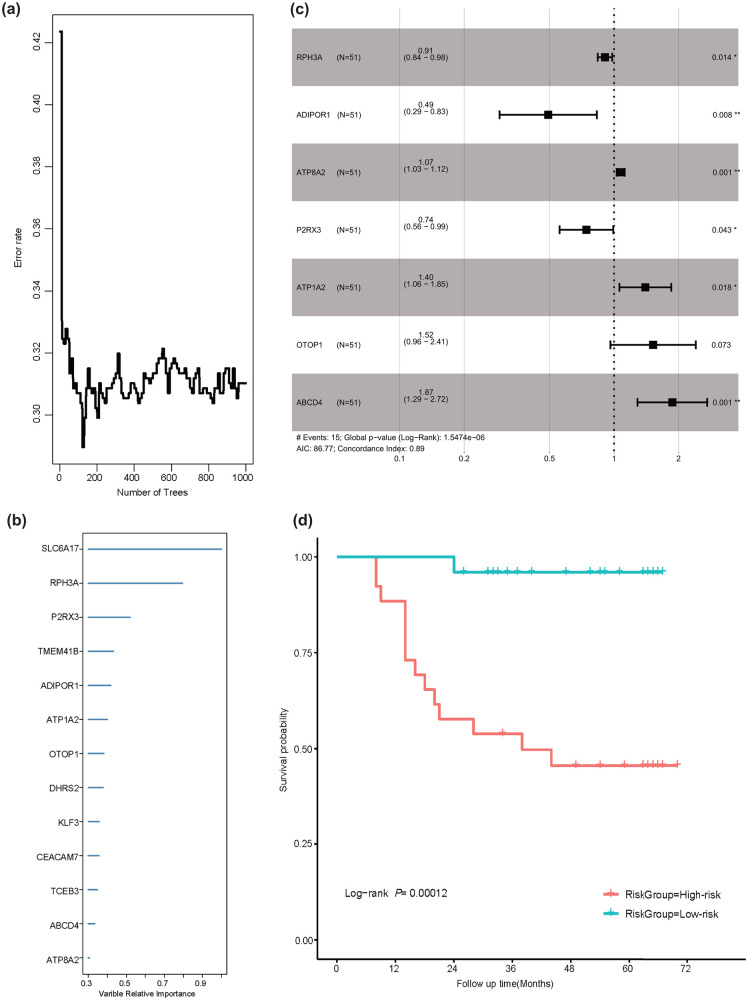
Marker genes selection and predicted model construction. (a. error rate curve of markers selection by RSF, **b.** Variable importance plot of genes with important value more than 0.5. **c.** Overall survival multivariate Cox regression analysis in GC patients. **d.** Kaplan-Meier plot of high and low risk-score groups, which separated using median risk score (1.28). RSF, random survival forest. HR, hazard ratio. CI, confidence interval.).

The random survival forest (RSF) model was applied to refine prognostic predictors. During model training, prediction error rates stabilized at low values after constructing 200 survival trees (Fig 2a). Variable importance (VIMP) scores were calculated upon completion of 1,000 survival trees, with higher VIMP indicating greater prognostic significance. Thirteen genes with VIMP > 0.3 were prioritized as candidate biomarkers (Fig 2b). Through stepwise multivariate Cox regression analysis, we refined these to seven core prognostic genes: RPH3A, ADIPOR1, ATP8A2, P2RX3, ATP1A2, OTOP1, and ABCD4. Multivariate Cox analysis confirmed the independent prognostic value of these seven genes (Fig 2c), with the resulting model demonstrating strong predictive accuracy (C-index = 0.892, 95% CI: 0.786–0.998). Using weighted 5hmC levels of the seven-gene signature, we calculated individualized risk scores. Patients were stratified into high-risk (n = 26) and low-risk (n = 25) groups based on the cohort median risk score (1.28). The high-risk group exhibited significantly worse overall survival compared to the low-risk group (log-rank P = 0.0001; Fig 2d).

**Fig 3:**
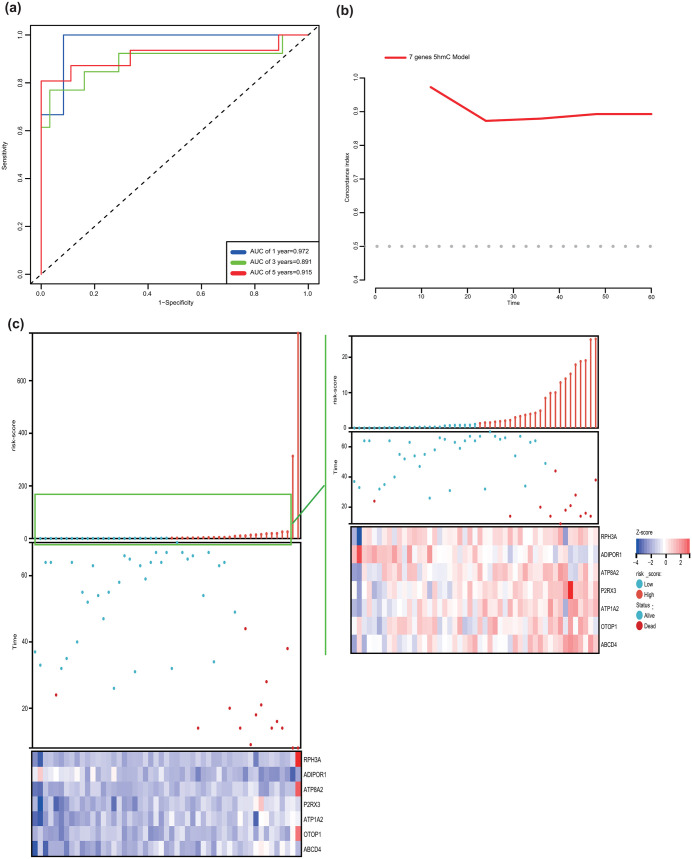
Risk-score calculated based on the seven biomarker genes 5hmC level are correlated with prognosis of GC patients (a. Time-dependent receiver operating characteristic (ROC) curves at 1 year,3years and 5 years. **b.** Time-dependent area under the curves (tdAUC). **c.** Linkage map of risk-score distribution and biomarker genes cluster).

Time-dependent receiver operating characteristic (ROC) curve analysis revealed strong prognostic performance of the risk score across multiple survival endpoints. The area under the curve (AUC) values for 1-, 3-, and 5-year overall survival predictions were 0.972, 0.891, and 0.912, respectively (Fig 3a). The time-dependent AUC (tdAUC) trajectory further demonstrated sustained predictive accuracy, maintaining values >0.8 throughout the 5-year observation period (Fig 3b). Risk score distribution patterns provided additional clinical insights. A combined visualization of risk score dynamics and 5hmC profile clustering showed that: Patients with risk scores above the cohort median (1.28) exhibited significantly shorter survival times, and two early mortality cases (8-month survival) demonstrated exceptionally elevated risk scores compared to the remainder of the cohort (Fig 3c).).

To evaluate the additive prognostic value of the 5hmC-derived risk score, we developed two Cox regression models. The Model 1 included baseline predictors (age, TNM stage, chemotherapy) selected through stepwise regression from clinical variables (sex, age, TNM stage, neural invasion, vascular invasion, Lauren classification, tumor size). Model 2 constructed by model 1 predictors + risk score. The C-index improved from 0.819 (95% CI: 0.727–0.911) for Model 1 to 0.904 (95% CI: 0.853–0.955) for Model 2. Multivariate analysis confirmed the risk score as an independent prognostic factor (HR = 27.47, 95% CI: 3.28–230.25; P < 0.001) after adjusting for age, TNM stage, and chemotherapy (Table 2).Model comparison using reclassification metrics demonstrated significant improvement with risk score inclusion: Net Reclassification Index (NRI) = 0.49 (95% CI: 0.12–0.76) and Integrated Discrimination Improvement (IDI)=0.25 (95% CI: 0.06–0.45).

**Table 2:** Multivariate prognostic models in patients with gastric cancer.

	Variable	*HR(95%CI)*	C-index(95%CI)
Model 1	TNM stage (III vs Ⅰ-Ⅱ)	13.71(2.97-63.32)	0.819(0.727-0.911)
	Chemotherapy(yes)	0.28(0.08-0.96)	
	Age(≥60)	1.68(0.53-5.33)	
Model 2	risk-score(high)	27.47(3.28-230.25)	0.904(0.853-0.955)
	TNM stage (III vs Ⅰ-Ⅱ)	23.11(4.24-126.03)	
	Chemotherapy(yes)	0.26(0.08-0.87)	
	Age(≥60)	3.79(1.07-12.43)	

**Fig 4:**
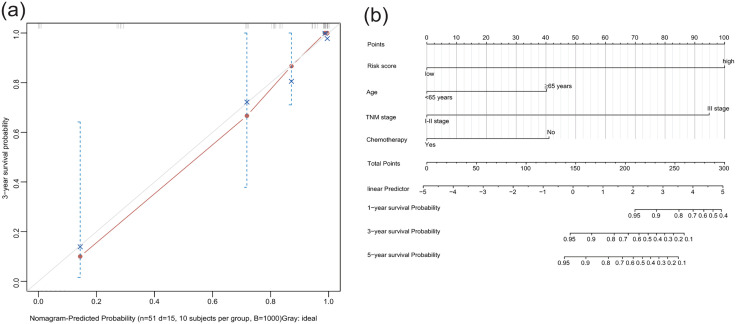
Calibrate curve and nomogram of the predicted model consisting risk-score with three characteristics (a. calibrate curve of model 2. b. nomogram of model 2 for predicting 1-,3- and 5 years survival in patients with GC. ).

The calibration curve for Model 2 exhibited excellent agreement between predicted and observed 5-year survival probabilities (Fig 4 a). We further developed a clinical nomogram integrating four predictors: age, TNM stage, chemotherapy status, and risk score for Model 2 (Fig 4 b). Each variable was assigned a point based on the HR value. Then, by summing the scores for each variable and locating the total score on the scale, the probabilities of 1-, 3-, and 5-year OS can be obtained. In the OS nomogram, the risk-score contributed the most to the survival outcome..

## 4. Discussion

The distinct genomic localization of 5hmC within transcriptionally active gene bodies, coupled with its strong correlation to transcriptional output, renders it a more robust epigenetic biomarker of disease progression than 5-methylcytosine (5mC) [22]. In this prospective cohort study of GC patients, we performed genome-wide 5hmC profiling of plasma-derived cfDNA to assess its prognostic utility. To our knowledge, this represents the first systematic evidence establishing genome-wide cfDNA 5hmC signatures as independent prognostic markers in GC. Through univariate Cox regression screening of 19,100 annotated genes, we identified 445 genes with differential hydroxymethylation significantly associated with clinical outcomes (P < 0.05). KEGG and GO functional enrichment analysis revealed these prognosis-linked genes were overrepresented in ‘tumor-related protein processing in the endoplasmic reticulum’, ‘cMAP signaling pathway’, ‘p53 signaling pathway’ and ‘transmembrane transporter complex’, ‘phosphoprotein binding’ gene ontology terms. These mechanisms are mechanistically linked to tumor progression through regulation of protein homeostasis, stress response signaling, and cellular transport dynamics—key hallmarks of cancer pathogenesis.

The RSF model represents a non-parametric, nonlinear ensemble learning technique that extends the conventional random forest methodology to accommodate survival data [23]. The RSF model is particularly suited for handling censored survival data, as it adapts the Gini impurity criterion by incorporating log-rank statistics to optimize node splits, thereby maximizing the divergence between Kaplan-Meier survival curves post-split [24]. RSF constructs multiple decision trees through a process of randomization and aggregation, culminating in a robust prediction model. Notably, the RSF approach does not rely on assumptions such as p-values, the proportional hazards assumption, or linearity, and reduces computational time by replacing cross-validation with out-of-bag data estimation [23]. RSF offers enhanced accuracy compared to alternative methodologies, presents a novel technical approach, and has provided valuable insights into the temporal variability of variable significance [25]. Through the application of RSF in conjunction with a stepwise Cox model, a panel of seven marker genes significantly associated with the prognosis of GC patients was identified, effectively combining the high predictive accuracy of the RSF model with the interpretative clarity of the Cox model. Notably, several of these biomarker genes, including ADIPOR1 (which mediates increased AMP-activated protein kinase and PPARα ligand activity, thereby negatively regulating cancer cell progression, have been previously implicated in GC [26]. The expression level of ADIPOR1 has also been reported to be associated with the development and progression of GC [27]. Additionally, some genes in the panel have been reported their specific role in other cancers but not in gastric cancer. For example, ATP8A2, which encoded ATPase Phospholipid Transporting 8A2, belongs to the P4-ATPase family. This gene family actively flips phosphatidylserine and phosphatidylethanolamine from the exoplasmic to the cytoplasmic leaflet of cell membranes to generate and maintain phospholipid asymmetry [28]. ATP8A2 has been associated with a better prognosis in lung cancer [29,30].

The risk score of the final predictive model integrating RSF and stepwise Cox analysis demonstrated robust performance in predicting GC prognosis, achieving a C-index of 89.1%, significantly superior to traditional TNM stage [31]. This enhanced efficacy is particularly noteworthy given that pathological TNM staging, while recognized as the gold standard for long-term survival prognostication in GC, can only be determined postoperatively [32]. Patients stratified into the high-risk group exhibited significantly worse OS compared to the low risk-score group (log-rank P = 0.00012). Importantly, the high-risk score remained independently associated with poor prognosis in multivariate analyses adjusted for TNM stage, age, and chemotherapy status, suggesting that 5hmC modification patterns in genes carry critical prognostic implications for GC. This finding aligns with prior studies demonstrating the prognostic value of 5hmC biomarkers in cfDNA across multiple cancer types [14,33–36]. The clinical relevance of such prognostic stratification is further underscored by evidence from other malignancies. For instance, treatment strategy optimization guided by prognostic biomarkers has improved survival outcomes in colorectal cancer [37]. As a treatment-agnostic biomarker capable of predicting OS regardless of therapeutic interventions [37], 5hmC-based risk stratification could inform clinical decision-making by identifying patients who may benefit from intensified surveillance or personalized adjuvant therapies.

Our analysis further revealed that integrating the cfDNA 5hmC epigenetic signature with conventional clinical prognostic parameters significantly improved prognostic discrimination accuracy. This synergistic combination demonstrated dual clinical utility: 1) as an autonomous prognostic biomarker independent of existing staging systems, and 2) as a complementary tool enhancing current risk stratification frameworks for GC. Notably, the composite model retained strong predictive capacity for long-term postoperative survival (C-index = 0.89; 95% CI 0.85–0.93) even after adjusting for therapeutic interventions, establishing 5hmC-based stratification as a treatment-agnostic prognostic determinant.

While this study provides novel insights into 5hmC-based prognostic stratification, several limitations should be acknowledged. The limitation of the small sample size in this study does not allow for the validation of the marker panel in independent samples. As the 5hmC prognostic signature was developed from a single site cohort, its validation in a broader population is warranted to ensure its applicability across varied clinical settings.

## Supporting information

S1 Table Top 30 enriched terms of KEGG analysis. (XLSX)

S2 Table Top 10 enriched terms of each gene function module of GO analysis. (XLSX)

S1 File Raw data of survival information. (XLSX)
